# Rapid Regression of a Hepatic Cyst Temporally Associated With Compensating Bio-Information Energy Modulation: A Speculative Case Report

**DOI:** 10.7759/cureus.102102

**Published:** 2026-01-22

**Authors:** Jia-Feng Yuan, Yong-De Chen, An-Na Xie, Xin-Zhou Yuan

**Affiliations:** 1 Research and Development Center, Shenzhen Apexflux Biotech Co. Ltd., Shenzhen, CHN

**Keywords:** case report, compensating bio-information energy, hepatic cyst, photon, spontaneous regression

## Abstract

Hepatic cysts are common benign liver lesions that usually remain stable, with spontaneous regression considered uncommon and slow. Novel non-invasive approaches that may favorably influence cyst resolution warrant exploration. We report a 75-year-old woman with a 4.8 cm cystic-solid lesion in hepatic segment IV identified during routine screening. The patient did not meet the criteria for interventional management but expressed psychological concern regarding disease progression. She underwent compensating bio-information energy (CBE) modulation, an approach using plant-derived ultra-weak photon emissions intended to promote physiological regulation, over an approximately eight-week period. Serial ultrasonography demonstrated progressive lesion regression, with measurable reduction at 17 days and complete radiological resolution by 10 weeks. No pharmacologic, surgical, or lifestyle interventions occurred concurrently. A 22-week consolidation follow-up confirmed durable resolution without recurrence, and the patient remained asymptomatic. The accelerated and sustained regression of a hepatic cyst temporally associated with CBE modulation contrasts with the slow natural history of spontaneous involution. Although causality cannot be established from a single case, this observation supports further controlled investigation into CBE as a potential non-invasive adjunct for benign hepatic cysts. While causality cannot be inferred from a single uncontrolled observation, this temporally associated regression should be regarded as a hypothesis-generating finding that warrants further investigation under rigorously controlled experimental and clinical conditions.

## Introduction

Hepatic cysts are benign, fluid-filled cavities within the liver parenchyma, typically lined by cuboidal or biliary epithelium and considered developmental, non-neoplastic lesions arising from aberrant biliary ductules [1]. With the increasing use of high-resolution abdominal ultrasonography, these lesions are now frequently detected incidentally, and population-based cohorts have reported a prevalence reaching up to 21.9%, with higher rates observed among older individuals and women [2].

Most hepatic cysts remain stable and asymptomatic throughout life, for which current clinical consensus recommends conservative management with periodic imaging surveillance [3]. Interventional procedures-such as percutaneous aspiration with sclerotherapy or laparoscopic fenestration-are reserved for large, symptomatic, or complicated cysts associated with pain, biliary or vascular compression, infection, or intracystic hemorrhage [4]. Although these procedures are generally effective, recurrence and procedure-related morbidity remain notable limitations [5]. For patients who do not meet interventional criteria, expectant observation remains the standard approach, though prolonged surveillance may impose psychological stress and financial burdens. Spontaneous regression of hepatic cysts has been documented but is rare and typically occurs gradually over several years [6]. Consequently, noninvasive approaches that might favorably modulate the hepatic microenvironment and promote cyst stability or resolution warrant systematic investigation.

Compensating bio-information energy (CBE) is a non-contact bio-informational approach that employs plant-derived ultra-weak photon emissions (UPE) to promote physiological self-regulation, which captures and amplifies plant UPE signals and converts them into a bio-informational field directed toward the human body [7,8]. These plant-origin informational fields are hypothesized to modulate biofield coherence, redox homeostasis, and neurohormonal balance through nonthermal mechanisms. Donor plants are selected based on their stable UPE intensity, metabolic vitality, and traditional relevance in phytomedicine, providing coherent and synergistic informational spectra [7]. Although mechanistic pathways remain hypothetical, CBE offers a structured framework for exploring biofield-mediated interactions between living systems.

The present report documents an atypical clinical observation involving a hepatic cyst that demonstrated an unusually rapid regression within a short time frame, deviating from the commonly described natural history of such lesions, following exposure to CBE modulation. While spontaneous regression remains the most scientifically grounded explanation, the accelerated course observed in this case is uncommon and warrants careful documentation. This report is therefore presented not to establish causality or therapeutic efficacy, but to provide a transparent, hypothesis-generating account of a temporally associated clinical observation.

## Case presentation

Baseline information

A 75-year-old woman underwent routine health screening in March 2023, during which abdominal ultrasonography identified a cystic-solid lesion in hepatic segment IV measuring approximately 4.8 × 3.7 × 4.6 cm. The liver appeared normal in size and morphology with a smooth capsule. The lesion demonstrated a predominantly hypoechoic echotexture with a central anechoic area, regular contour, and relatively well-defined margins. Color Doppler flow imaging (CDFI) revealed peripheral strip-like vascular signals without abnormal intraparenchymal flow. The intrahepatic ducts and main portal vein were of normal caliber. The impression was a cystic-solid nodule, likely benign, for which percutaneous biopsy was suggested.

Given the lesion size and absence of clinical symptoms, the patient did not meet interventional criteria and was managed conservatively in accordance with standard clinical guidelines. Nevertheless, the patient reported persistent psychological concern about potential lesion progression and sought a noninvasive wellness intervention.

At baseline, contrast-enhanced imaging modalities such as computed tomography or magnetic resonance imaging were discussed as potential options for further lesion characterization. However, given the benign ultrasonographic appearance, absence of clinical symptoms, and the patient’s preference for conservative management, additional contrast-enhanced imaging was not pursued, and close serial ultrasonographic follow-up was selected.

Device description and intervention protocol

**Figure 1:**
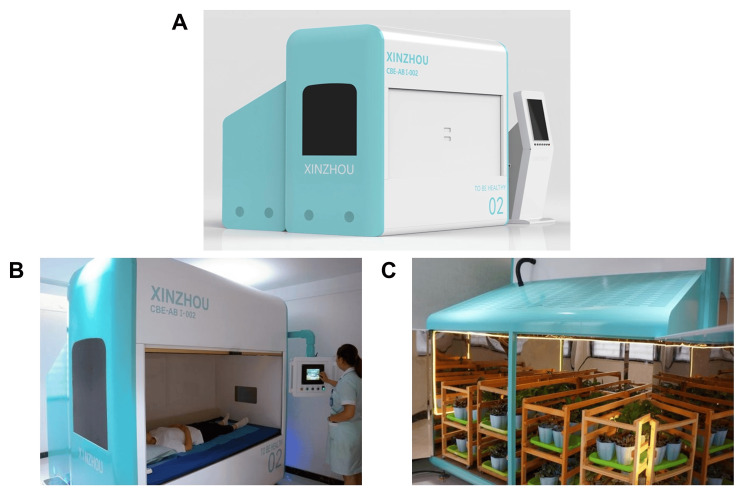
Bio-information self-healing cabin for compensating bio-information energy (CBE) modulation. (A) External view of the complete cabin system. (B) Human reception chamber with participant bed and emission plate. (C) Plant signal acquisition chamber with donor plant racks.

The patient consented to receive CBE modulation using a bio-information self-healing and rehabilitation cabin (BIC-A30-S-001, Apexflux Biotech, Shenzhen, China), a patented system (Patent No. 201020667266.9) (Figure 1A). It is important to note that the biophysical principles underlying CBE remain highly speculative and are not supported by mainstream scientific consensus. Accordingly, the following description reflects the conceptual framework and claims proposed by the developers and is provided for descriptive purposes only, without implying established scientific validity or clinical efficacy. The cited patent number refers solely to intellectual property protection and does not constitute evidence of scientific validation or therapeutic effectiveness.

According to the developer’s description, the device comprises two integrated modules: (A) a plant signal acquisition chamber, in which donor plants are placed on dedicated racks and are claimed to generate UPE (Figure 1C); and (B) a human reception chamber, in which the subject rests on a specialized bed. The developer describes a low-energy linear particle accelerator located beneath the bed that interfaces with a signal-transfer conduit, which is claimed to deliver an amplified plant-derived informational field to an emission plate situated above the bed, thereby exposing the participant to what is described as a nonthermal bio-informational field (Figure 1B). Donor material consisted of 15 × 4 trays of live sprouting plants per session, selected by the developer for high metabolic vitality and stable UPE characteristics. According to the developer, the system does not emit electromagnetic radiation beyond ambient environmental background levels and does not generate thermal, mechanical, or electrical stimulation.

Sessions were scheduled as three exposures per day, each lasting 40 minutes, with the participant supine within the reception chamber for the duration of each exposure. The CBE regimen commenced in March 2023 and was planned for 17 calendar days; in practice, two days were missed irregularly during this initial interval (resulting in 15 effective exposure days). Thereafter, modulation continued on a daily basis, with infrequent absences (approximately two days per month), until June 2023, yielding an aggregate exposure period of approximately eight weeks. No concomitant pharmacologic, surgical, or physical therapies were administered during the observation period.

Results

**Figure 2:**
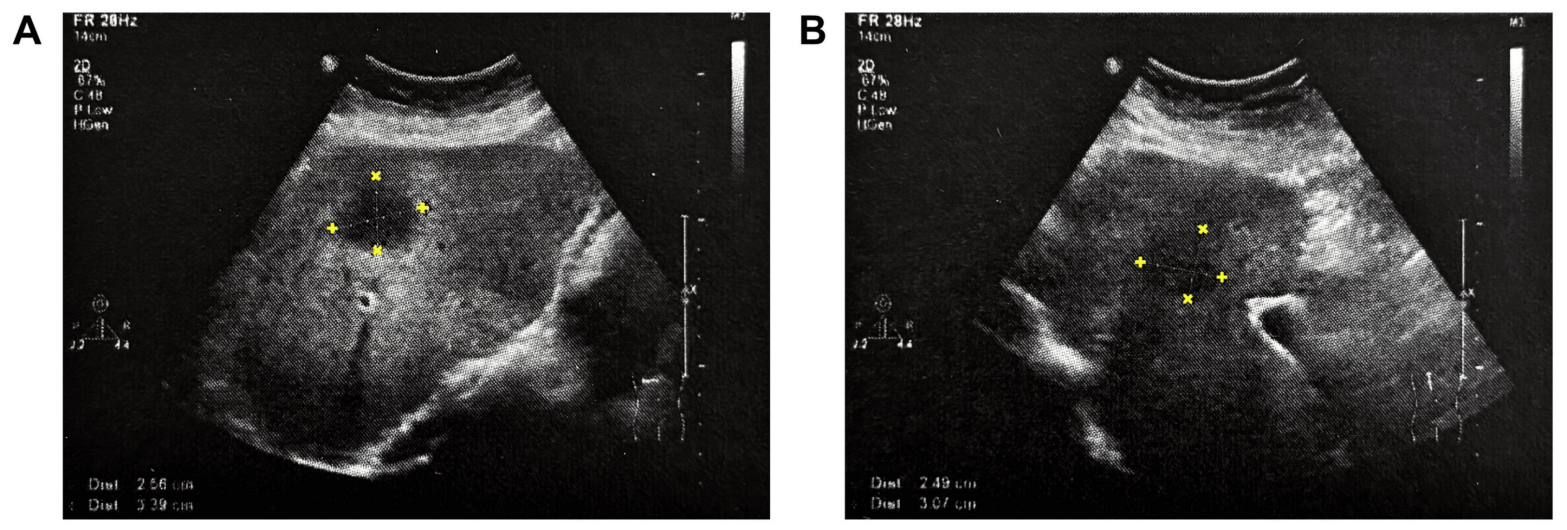
Hepatobiliary-pancreatic-splenic and portal venous color Doppler ultrasonography in April 2023 (17 days after initiation of compensating bio-information energy (CBE) wellness modulation).

A follow-up hepatobiliary-pancreatic-splenic ultrasound performed in April 2023, after the initial 17-day interval of the CBE program (15 effective exposure days), demonstrated a reduction in lesion size to 3.1 × 3.4 × 2.6 cm and a shift toward a predominantly solid echotexture (Figure 2). The nodule retained a regular contour with mildly indistinct margins and exerted only a minimal space-occupying effect. There was no biliary dilatation or abnormal intraparenchymal flow on CDFI. The findings suggested progressive regression of the lesion relative to the baseline study.

**Figure 3:**
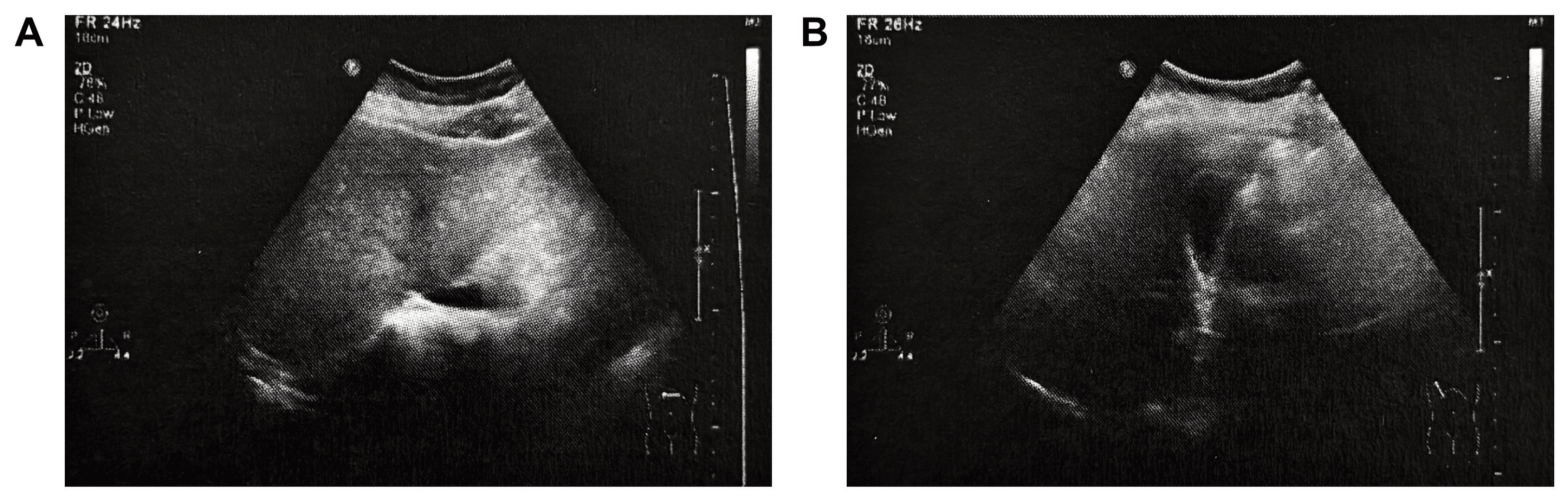
Hepatobiliary-pancreatic-splenic and portal venous color Doppler ultrasonography in June 2023 (after 10 weeks of compensating bio-information energy (CBE) modulation).

By June 2023, following approximately ten weeks of cumulative CBE exposure, repeat ultrasonography demonstrated a normal-sized liver with no detectable cystic or solid lesion in segment IV (Figure 3). The hepatic parenchyma showed mildly increased echogenicity consistent with mild hepatic steatosis, but no residual focal lesion or abnormal vascular flow was identified. The finding suggested complete radiological resolution of the prior cystic-solid lesion.

**Figure 4:**
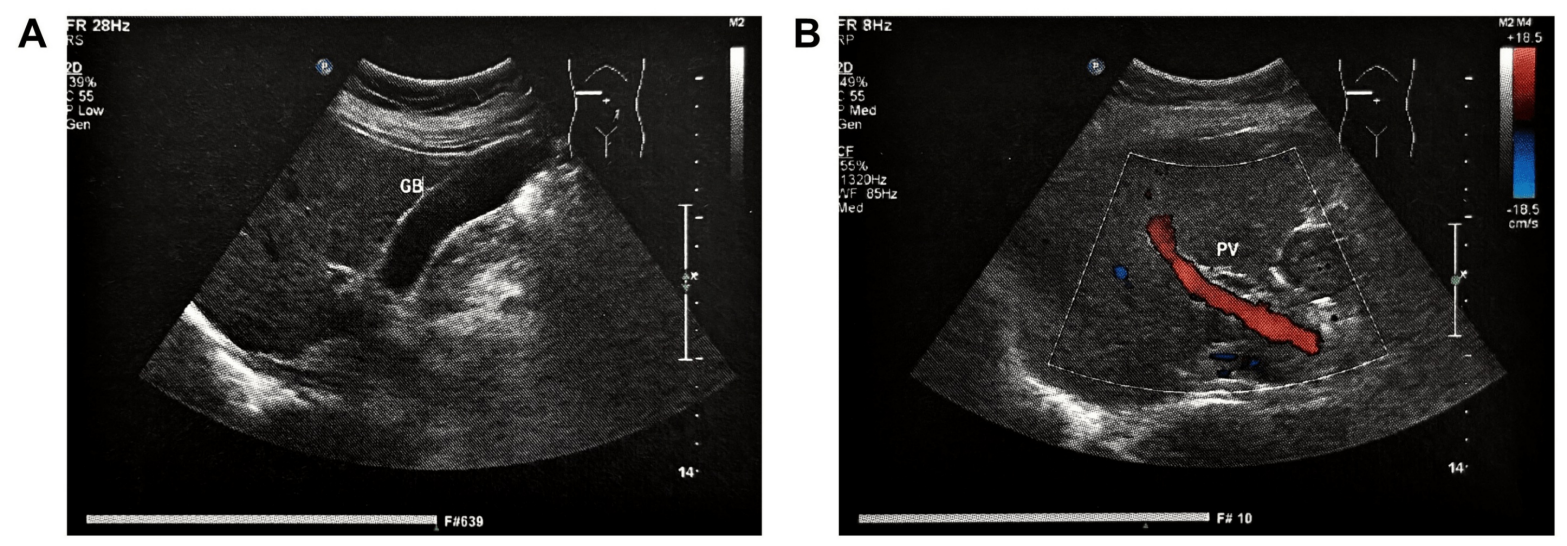
Hepatobiliary-pancreatic-splenic and portal venous color Doppler ultrasonography in December 2023 (22 weeks after lesion resolution and consolidation follow-up of compensating bio-information energy (CBE) modulation).

A consolidation ultrasound performed in December 2023 (22 weeks after documented resolution) confirmed stable hepatic architecture without recurrence (Figure 4). The liver capsule remained smooth, the intrahepatic ducts and main portal vein were normal in caliber, and mild diffuse fatty infiltration persisted. The gallbladder, pancreas, and spleen were unremarkable.

Across sequential ultrasonographic evaluations performed using the same imaging modality, the hepatic lesion demonstrated a progressive pattern of regression, with early size reduction followed by complete radiological disappearance within approximately 10 weeks of cumulative exposure. Subsequent consolidation follow-up extending over several additional months confirmed stable hepatic architecture without evidence of lesion recurrence. No new focal hepatic lesions, biliary dilatation, or abnormal intraparenchymal vascular signals were identified, and the patient remained clinically asymptomatic throughout the observation period without requiring any pharmacologic, surgical, or interventional management.

## Discussion

This case report documents the complete radiological resolution of a hepatic cyst following CBE modulation. From the standpoint of established clinical knowledge, spontaneous regression remains the most parsimonious and scientifically grounded explanation for changes observed in benign hepatic cysts, particularly in the absence of pharmacologic or interventional treatment. Simple hepatic cysts are generally considered stable lesions with minimal short-term dimensional change, and while spontaneous regression has been reported, it is regarded as uncommon and typically unfolds over extended durations [9]. For example, Arai et al. reported an isolated case in which a 7.7 cm cyst regressed to 1.0 cm over approximately eight years [6], and Tsuruya et al. observed cyst regression or partial involution in 26.6% of individuals in a 10-year population-based follow-up, with most cases demonstrating slow volume reduction and the emergence of hyperechoic intracystic fluid, suggesting gradual resorption rather than abrupt collapse [2].

In contrast, the present patient exhibited measurable lesion reduction within 17 days and complete radiological resolution within approximately 10 weeks, with sustained stability observed at a 22-week follow-up visit. Given that no pharmacologic, surgical, or physical treatment was administered during this period and lifestyle factors remained stable, careful documentation of this accelerated involution is warranted, while acknowledging the inherent uncertainty regarding causation [5,9]. The temporal association between CBE exposure and lesion regression, therefore, invites cautious discussion of potential biological considerations and methodological limitations, without implying therapeutic efficacy.

UPE refers to spontaneous photon emission from living organisms, typically within the 200-800 nm spectral range, arising from oxidative metabolic processes and electronically excited biomolecular states [10]. In plant systems, UPE correlates with cellular redox dynamics and metabolic activity and has been proposed by some investigators to contribute to biological information transfer [11,12]. Building on these principles, the CBE system has been developed to capture, amplify, and transmit plant-derived UPE signals using a low-energy linear accelerator, with the aim of generating a structured bio-informational field directed toward the human body [8]. Donor plants are selected based on UPE stability, metabolic vitality, and established phytotherapeutic relevance, with multi-species combinations configured to optimize spectral coherence and informational diversity [7].

From a biophysical perspective, UPE is closely linked to mitochondrial metabolism and reactive oxygen species (ROS) generation [10,11]. Independent of CBE, a substantial body of experimental research in photobiomodulation has demonstrated that low-intensity photons within overlapping spectral ranges can influence cytochrome-c-oxidase activity, mitochondrial membrane potential, ATP synthesis, and redox balance under controlled laboratory conditions [13-16]. These observations represent established findings in cellular bioenergetics; however, their applicability to externally derived, plant-origin photonic signals in human clinical settings remains unverified.

Beyond mitochondrial bioenergetics, emerging theoretical models have proposed that UPE may contribute to long-range intracellular coordination through resonance energy transfer within mitochondrial-microtubule networks [17,18]. Intracellular chromophores, including NAD(P)H and components of the electron transport chain, are capable of absorbing and re-emitting photons, forming what has been described as an “organic photonic network” that could hypothetically support coordinated redox signaling. Additionally, UPE has been detected in neuronal systems, with emission intensity increasing during metabolic activation and decreasing following sodium-channel blockade, suggesting a potential relationship between photonic activity and neuro-metabolic coupling [19,20]. While these findings support the plausibility of endogenous photonic signaling, extrapolation to plant-derived UPE-mediated modulation in human tissues remains speculative.

In the context of hepatic cyst pathology, where cyst-wall epithelial metabolic regulation, redox balance, and interstitial fluid dynamics are thought to influence lesion persistence or involution, proponents of CBE have hypothesized that bio-informational modulation could potentially influence epithelial transport processes or microvascular coordination. Nevertheless, such interpretations remain unproven, and alternative explanations, including coincidental alignment with an uncommon but naturally occurring rapid spontaneous regression, cannot be excluded.

Taken together, while spontaneous regression remains the most scientifically grounded explanation for the lesion resolution observed in this case, the unusually rapid time course and its temporal coincidence with CBE exposure justify cautious documentation as an atypical clinical observation. Rather than supporting clinical application, this finding suggests potential research interest in bio-informational modulation as a theoretical construct, but rather underscores the need for rigorously designed, blinded, placebo-controlled studies and independently reproducible mechanistic investigations before such approaches can be meaningfully evaluated in clinical practice.

## Conclusions

While causality cannot be inferred from a single case report, the accelerated and sustained regression observed in this patient deviates from the typical natural history of simple hepatic cysts and merits cautious scientific attention. The temporal relationship between CBE exposure and lesion regression, in the absence of concurrent medical interventions, supports consideration of this observation within an exploratory and hypothesis-generating framework. This report describes a temporal association observed in a single uncontrolled case and, while not implying causality, highlights both the potential research interest and the substantial methodological challenges involved in scientifically evaluating unconventional and non-mainstream bio-informational claims.

## References

[1] Borhani AA, Wiant A, Heller MT (2014). Cystic hepatic lesions: a review and an algorithmic approach. AJR Am J Roentgenol.

[2] Tsuruya K, Nishizaki Y, Tatemichi M (2022). The prevalence and natural history of hepatic cysts examined by ultrasound: a health checkup population retrospective cohort study. Sci Rep.

[3] Shimizu T, Yoshioka M, Kaneya Y (2022). Management of simple hepatic cyst. J Nippon Med Sch.

[4] Vardakostas D, Damaskos C, Garmpis N, Antoniou EA, Kontzoglou K, Kouraklis G, Dimitroulis D (2018). Minimally invasive management of hepatic cysts: indications and complications. Eur Rev Med Pharmacol Sci.

[5] Furumaya A, van Rosmalen BV, de Graeff JJ (2021). Systematic review on percutaneous aspiration and sclerotherapy versus surgery in symptomatic simple hepatic cysts. HPB (Oxford).

[6] Arai H, Nagamine T, Suzuki H (2002). Simple liver cyst with spontaneous regression. J Gastroenterol.

[7] Yuan X, Yuan J, Deng Z (2022). The experimental exploration and discovery of DNA communication between plants. JMP.

[8] Yuan X, Yuan J, Bi Q (2022). Study on plant radiation signal transduction for human self-healing. Open J Biophys.

[9] Drenth J, Barten T, Hartog H (2022). EASL Clinical Practice Guidelines on the management of cystic liver diseases. J Hepatol.

[10] Mould RR, Mackenzie AM, Kalampouka I, Nunn AV, Thomas EL, Bell JD, Botchway SW (2024). Ultra weak photon emission—a brief review. Front Physiol.

[11] Volodyaev I, Beloussov LV (2015). Revisiting the mitogenetic effect of ultra-weak photon emission. Front Physiol.

[12] Benfatto M, Pace E, Davoli I (2023). Biophotons: new experimental data and analysis. Entropy (Basel).

[13] Hamblin MR (2017). Mechanisms and applications of the anti-inflammatory effects of photobiomodulation. AIMS Biophys.

[14] de Freitas LF, Hamblin MR (2016). Proposed mechanisms of photobiomodulation or low-level light therapy. IEEE J Sel Top Quantum Electron.

[15] Hamblin MR (2018). Mechanisms and mitochondrial redox signaling in photobiomodulation. Photochem Photobiol.

[16] Ferraresi C, Kaippert B, Avci P (2015). Low-level laser (light) therapy increases mitochondrial membrane potential and ATP synthesis in C2C12 myotubes with a peak response at 3-6 h. Photochem Photobiol.

[17] Thar R, Kühl M (2004). Propagation of electromagnetic radiation in mitochondria?. J Theor Biol.

[18] McFadden J, Al-Khalili J (2018). The origins of quantum biology. Proc Math Phys Eng Sci.

[19] Esmaeilpour T, Fereydouni E, Dehghani F (2020). An experimental investigation of ultraweak photon emission from adult murine neural stem cells. Sci Rep.

[20] Tang R, Dai J (2014). Spatiotemporal imaging of glutamate-induced biophotonic activities and transmission in neural circuits. PLoS One.

